# Associations of albumin and nutritional index factors with delirium in patients admitted to the cardiac intensive care unit

**DOI:** 10.3389/fcvm.2023.1100160

**Published:** 2023-03-03

**Authors:** Tae Wan Kim, Ryoung Eun Ko, Soo Jin Na, Chi Ryang Chung, Ki Hong Choi, Chi-Min Park, Jeong Hoon Yang

**Affiliations:** ^1^Department of Critical Care Medicine, Samsung Medical Center, Sungkyunkwan University School of Medicine, Seoul, Republic of Korea; ^2^Division of Cardiology, Department of Medicine, Heart Vascular Stroke Institute, Samsung Medical Center, Sungkyunkwan University School of Medicine, Seoul, Republic of Korea

**Keywords:** delirium, albumin, malnutrition, cardiac intensive care unit, nutrition indexes

## Abstract

**Background:**

Limited data are available on the association of malnutrition with the occurrence of delirium in the cardiac intensive care unit (CICU). Thus, we aimed to analyze whether nutritional indices and their components can predict the development of delirium in CICU.

**Methods:**

We enrolled 2,783 patients admitted to the CICU of Samsung Medical Center for more than 24 h between September 2012 and December 2018. We assessed the nutritional status at admission using three indices, the Prognostic Nutrition Index (PNI), the Geriatric Nutritional Risk Index (GNRI), and the Controlling Nutritional Status (CONUT). Then, we compared predictive performances for the occurrence of delirium among nutritional indices using Delong’s test.

**Results:**

Delirium developed in 678 patients (24.3%) assessed three times daily for 7 days of CICU stay. Nutritional indices had fair predictive performance for development of delirium in critically ill cardiac patients using the area under the receiver-operating characteristic curve (AUROC: 0.729 for the GNRI, 0.728 for PNI, and 0.762 for CONUT). Furthermore, the AUROC of albumin alone (0.77, 95% CI, 0.75–0.79) was significantly greater than that of either GNRI (*p <* 0.001) or PNI (*p <* 0.001). In a multivariable analysis including each component of nutritional indices, albumin was a significant predictor for delirium but not absolute lymphocyte count, bodyweight/ideal bodyweight, or total cholesterol level as a component of nutritional indices.

**Conclusion:**

Predictive performances of nutritional indices for the occurrence of delirium were acceptable in patients admitted to CICU. Albumin alone might be a helpful and straightforward indicator for the occurrence of delirium.

## Introduction

Delirium is a frequent complication in patients admitted to the intensive care unit (ICU). It has been associated with increased risk of mortality, development of post-ICU cognitive impairment, and longer ICU and hospital stays (1, 2). In recent decades, the application of advanced medical devices for the treatment of cardiovascular disease has been significantly expanded, and the number of critically ill patients with underlying cardiac disease has increased (3). Accordingly, the incidence of delirium in the cardiac intensive care unit (CICU) has substantially increased and is comparable to that in other medical ICUs (4). Furthermore, recent studies have reported associations between delirium and clinical outcomes in critically ill cardiac patients (5, 6).

Malnutrition is an essential risk factor for delirium in elderly patients (7). Several nutritional indices have been developed to assess the risk of malnutrition in critically ill patients. First, the Prognostic Nutritional Index (PNI) was created in 1980 to evaluate preoperative nutritional conditions and surgical risk for gastrointestinal surgery (8). The Geriatric Nutritional Risk Index (GNRI) was developed to estimate the risk of morbidity and mortality in elderly medical patients (9). The Controlling Nutritional Status (CONUT) is widely used for daily assessment of the nutritional status of inpatients (10).

Although several studies have reported an association of delirium with mortality in ICU patients, limited studies are available on the relationship between nutritional status and delirium in CICU patients. Therefore, the goal of the present study was (i) to assess the predictive performance of nutritional indices (PNI, GNRI, and CONUT) and their components in CICU patients and (ii) to investigate clinical factors associated with delirium in this population.

## Methods

### Study population

All consecutive patients who were admitted to the CICU between September 1, 2012, and December 31, 2018, were eligible for the study. A total of 4,261 patients who were 18 years old or older was admitted to the CICU at Samsung Medical Center in Seoul, Republic of Korea. We excluded 1,473 patients who stayed in the CICU for less than 24 h and three patients for whom we were not able to calculate nutritional indices due to limited data. The Institutional Review Board of Samsung Medical Center approved this study (IRB. No. 2020–10-102) and waived the requirement for informed consent because of the observational nature of the study. Patient information was anonymized and de-identified before analysis.

### Cardiac intensive care unit management

Our unit is a 12-bed ICU with a 1:2 nurse-to-patient ratio offering level 1 care for critically ill cardiac patients. To provide comprehensive critical care to patients with various cardiovascular diseases and complex comorbidities, the CICU is equipped with invasive and noninvasive devices to monitor patients’ hemodynamic status and provide advanced therapeutic technologies. With the high-intensity staffing model, patients are managed by a dedicated cardiac intensivist who is board certified in interventional cardiology and critical care medicine (11). Cardiac surgery support is promptly assessable. In addition, multidisciplinary care is provided *via* consultation with a dietitian, pharmacist, and respiratory care practitioner.

The clinical practice guidelines published by The Society of Critical Care Medicine were adopted for general intensive care (12). The Confusion Assessment Method for ICU (CAM-ICU) assessment was performed by nurses three times a day on patients with a Richmond Agitation-Sedation Scale of −3 (indicating movement or eye-opening to voice but no eye contact) or higher. The recorded CAM-ICU results were re-checked every day by a senior nurse. Nutrition support team consultation was available for nutrition therapy.

### Nutritional indices

Nutritional status was assessed using the PNI, GNRI, and CONUT based on blood test results measured on the day of admission to CICU to identify risk scores of malnutrition in critically ill patients. The PNI is calculated as follows: 10 x serum albumin concentration (g/dL) + 0.005 x absolute lymphocyte count (ALC, number/mm^2^) in peripheral blood (13). Patients with a PNI score > 38 were considered normal, patients with a score of 35–38 were at risk of moderate malnutrition, and patients with a score < 35 were classified as at risk of severe malnutrition. The GNRI is calculated as follows: 14.89 x serum albumin (g/dL) + 41.7 x [bodyweight/ideal bodyweight (IBW)] (9). Patients were categorized into four groups according to GNRI score: major risk (GNRI score < 82), moderate risk (82 ≤ GNRI score < 92), low risk (92 ≤ GNRI score < 98), and no risk (98 ≤ GNRI score). The CONUT was obtained based on serum albumin concentration, cholesterol level, and ALC (14). According to the CONUT score, patients were divided into four groups: normal (0–1 point), mild risk (2–4 points), moderate risk (5–8 points), and severe risk (9–12 points).

### Data collection

After reviewing the medical records, a trained study coordinator retrospectively collected clinical, laboratory, and outcome data. To accurately evaluate the total number of days at risk of delirium, we did not include days when the patient was in a coma as defined by a Richmond Agitation-Sedation Scale assessment of −4 (unresponsive to voice but responded to physical stimulation) or − 5 (unresponsive to voice and physical stimulation) (15). Delirium was defined as CAM-ICU positivity at any time during the assessment three times daily for up to 7 days of CICU stay. Polypharmacy was defined as the routine use of five or more medications (16).

### Statistical analysis

Descriptive statistics were performed to compare the clinical characteristics and outcomes between the delirium and non-delirium groups. Continuous variables were presented as median and interquartile range or mean ± standard deviation and were compared with a Mann–Whitney *U* test. Categorical variables were expressed as number and percentage and were analyzed using Chi-square tests or Fisher’s exact tests, where applicable. The model’s predictive ability to discriminate between patients who had and did not have delirium was estimated using the receiver operating characteristic curve. The receiver operating characteristic curve is presented to demonstrate the performance of delirium for the area under the curve (AUROC) and 95% confidence interval (CI). Discriminant functions were compared by a 2-tailed paired comparison of the ROC curve by DeLong’s method (17). Variables with a *p* value less than 0.05 on univariate analyzes and clinically relevant *a priori* variables were entered into the multiple logistic regression model. Results were reported as each variable’s odds ratio (OR) with 95% CI. A two-tailed *p* value less than 0.05 was considered statistically significant for all analyzes. Data were analyzed using R Statistical Software (Version 3.2.5; R Foundation for Statistical Computing, Vienna, Austria).

## Results

### Clinical characteristics

During the study period, 2,783 patients were finally enrolled in this analysis; 678 patients (24.3%) have occurred delirium during CICU admission. The characteristics of the patients are shown in Table 1. Patients in the delirium group were older and higher-risk subjects with lower body mass index and more comorbidities than those in the non-delirium group. There was no difference between the two groups in the CICU admission diagnosis and polypharmacy. Cardiac arrest before CICU admission (20.9% vs. 6.3%; *p <* 0.001) and Sequential Organ Failure Assessment score (6.97 ± 3.47 vs. 3.39 ± 2.71; *p <* 0.001) were higher in the delirium group than in the non-delirium group. In the laboratory findings at CICU admission, ALC [1.13 (0.7–1.8) x10^3^/μL vs. 1.62 (1.1–2.3) x10^3^/μL; *p <* 0.001], albumin [3.2 (2.8–3.6) g/dL vs. 3.9 (3.4–4.2) g/dL; *p <* 0.001], and total cholesterol [119.5 (94.0–152.0) mg/dL vs. 150.0 (121.5–183.0) mg/dL; *p <* 0.001] were significantly lower in the delirium group.

**Table 1:** Baseline characteristics of CICU patients.

	Delirium (*n =* 678)	Non-delirium (*n =* 2,105)	*P* value
Age, years	69.25 ± 14.7	63.24 ± 14.7	<0.001
Sex, male	384 (56.6)	1,416 (67.3)	<0.001
Body mass index, kg/m^2^	23.29 ± 4.08	24.04 ± 3.80	<0.001
Comorbidities			
Hypertension	417 (61.5)	1,127 (53.5)	<0.001
Diabetes mellitus	265 (39.1)	637 (30.3)	<0.001
Previous coronary disease	214 (31.6)	509 (24.2)	<0.001
Chronic kidney disease^*^	34 (5.0)	81 (3.8)	0.184
Stroke	154 (22.7)	213 (10.1)	<0.001
Dementia	30 (4.4)	11 (0.5)	<0.001
Smoking	120 (17.8)	477 (22.7)	0.008
Reason for CICU admission			0.257
Acute coronary syndrome	332 (49.0)	1,006 (47.8)	
Heart failure	164 (24.2)	574 (27.3)	
Arrhythmia	96 (14.2)	230 (10.9)	
Aortic disease	33 (4.9)	125 (5.9)	
Pulmonary hypertension	28 (4.1)	88 (4.2)	
Pericardial disease	22 (3.2)	64 (3.0)	
Others	1 (0.1)	10 (0.5)	
Admission route			<0.001
Emergency room	304 (44.8)	1,340 (63.7)	
General ward	143 (21.1)	260 (12.4)	
Outpatient department	127 (18.7)	161 (7.6)	
Other intensive care units	2 (0.3)	2 (0.1)	
Polypharmacy^**^	94 (13.9)	350 (16.6)	0.099
Cardiac arrest before CICU admission	142 (20.9)	133 (6.3)	<0.001
SOFA score at CICU admission	6.97 (±3.47)	3.39 (±2.71)	<0.001
Laboratory findings			
White blood cell count, 10^3^/μL	12.92 (9.7–17.1)	9.21 (7.1–12.4)	<0.001
ALC, x10^3^/μL	1.13 (0.7–1.8)	1.62 (1.1–2.3)	<0.001
Hemoglobin, g/dL	10.10 (8.8–11.8)	12.40 (10.5–14.0)	<0.001
Platelet, x10^3^/μL	146.0 (93.0–207.0)	188.0 (146.0–230.0)	<0.001
Total bilirubin, mg/dL	1.10 (0.7–2.0)	0.90 (0.6–1.3)	<0.001
Albumin, g/dL	3.20 (2.8–3.6)	3.90 (3.4–4.2)	<0.001
Blood urea nitrogen, mg/dL	29.40 (20.5–46.9)	19.10 (14.2–28.3)	<0.001
Creatinine, mg/dL	1.48 (1.0–2.3)	1.01 (0.8–1.4)	<0.001
Total cholesterol, mg/dL	119.5 (94.0–152.0)	150.0 (121.5–183.0)	<0.001
LDL cholesterol, mg/dL	73.00 (49.0–107.0)	98.00 (73.0–126.0)	<0.001
HDL cholesterol, mg/dL	38.00 (30.0–48.0)	44.00 (36.0–53.0)	<0.001
Triglycerides, mg/dL	99.0 (70.0–135.0)	106.0 (75.0–155.0)	0.004
Troponin I, ng/mL	1.75 (0.2–18.4)	1.00 (0.1–18.0)	0.002
NT–pro BNP, pg./mL	7, 428(1,856–18,114)	1,388(246–6,286)	<0.001
C–reactive protein, mg/dL	4.0 (0.9–10.1)	0.5 (0.1–3.3)	<0.001
Serum glucose maximum, mg/dL	169.0 (121.0–230.0)	135.0 (113.0–178.5)	<0.001

Values are the mean ± standard deviation, median (interquartile range), or *n* (%). ^*^Chronic kidney disease is defined as end-stage renal disease on hemodialysis or peritoneal dialysis. ^**^Polypharmacy is defined as the routine use of five or more medications. ALC, absolute lymphocyte count; CICU, cardiac intensive care unit; HDL, high density lipoprotein; LDL, low density lipoprotein; NT pro-BNP, N-terminal pro-brain natriuretic peptide; SOFA score, Sequential Organ Failure Assessment score.

### Cardiac intensive care unit management and clinical outcomes

Patients in the delirium group were more likely to receive vasopressor, mechanical ventilator, intraaortic balloon pump, and extracorporeal membrane oxygenator interventions than were those in the non-delirium group during the CICU stay (Table 2). Compared with the non-delirium group, CICU length of stay (8.15 ± 11.35 days vs. 2.88 ± 3.52 days; *p <* 0.001), CICU mortality (11.4% vs. 1.8%; *p <* 0.001), and in-hospital mortality (18.3% vs. 3.1%; *p <* 0.001) were higher in the delirium group.

**Table 2:** In-hospital management and clinical outcomes.

	Delirium (*n =* 678)	Non–delirium (*n =* 2,105)	*P* value
In-hospital management			
Vasopressor	360 (53.1)	467 (22.2)	<0.001
Mechanical ventilation	346 (51.0)	520 (24.7)	<0.001
Renal replacement therapy	57 (8.4)	208 (9.9)	0.285
Intraaortic balloon pump	32 (4.7)	47 (2.2)	<0.001
ECMO	138 (20.4)	179 (8.5)	<0.001
Medical events during CICU			
Sepsis	22 (3.2)	13 (0.6)	<0.001
Bleeding	7 (1.0)	7 (0.3)	0.054
Stroke	11 (1.6)	3 (0.1)	<0.001
Clinical outcomes			
Length of stay in CICU, days	8.15 (± 11.35)	2.88 (± 3.52)	<0.001
CICU mortality	77 (11.4)	38 (1.8)	<0.001
In-hospital mortality	124 (18.3)	65 (3.1)	<0.001

Values are the mean ± standard deviation, median (interquartile range), or *n* (%). CICU, cardiac intensive care unit; ECMO, extracorporeal membrane oxygenation.

### Predictive performance for delirium

**Figure 1:**
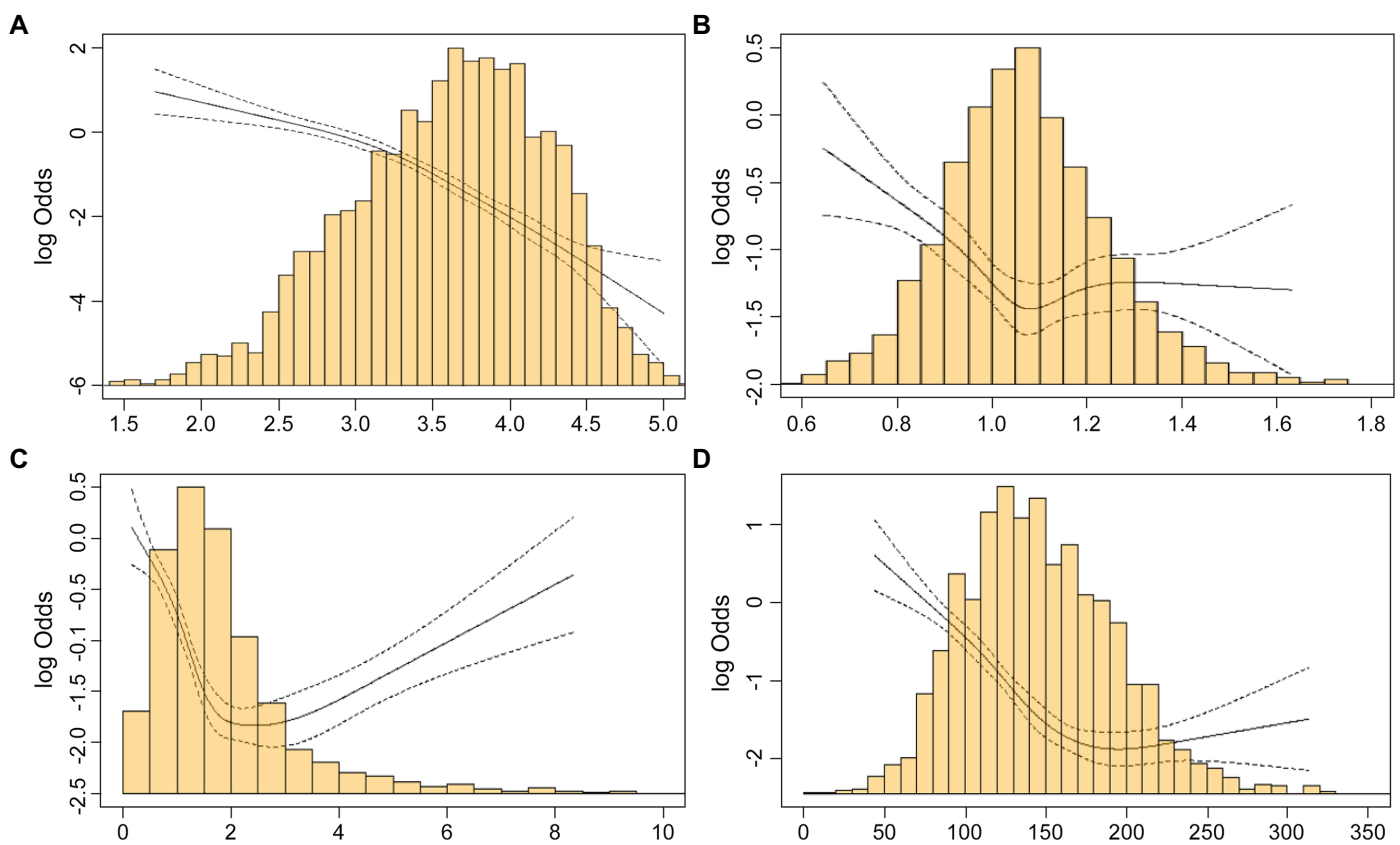
Restricted cubic spline plot of the relationships between components of nutrition indices and delirium. The solid line is the log odds, and the dashed lines are the upper and lower 95% confidence limits. **(A)** Restricted cubic spline plot for the association between albumin (g/dL) and delirium. **(B)** Restricted cubic spline for the association between bodyweight/ideal bodyweight and delirium. **(C)** Restricted cubic spline for the association between absolute lymphocyte count (x10^3^/μL) and delirium. **(D)** Restricted cubic spline for the association between total cholesterol (ml/dL) and delirium.

The distribution of delirium according to the value of each component in the three nutritional indices is shown in cubic spline curves (Figure 1). According to the increasing level of albumin, the occurrence of delirium tended to decrease linearly (Figure 1A). However, U-shaped correlation was observed for increased level of ALC, bodyweight/IBW, and total cholesterol with delirium (Figures 1B–D).

**Figure 2:**
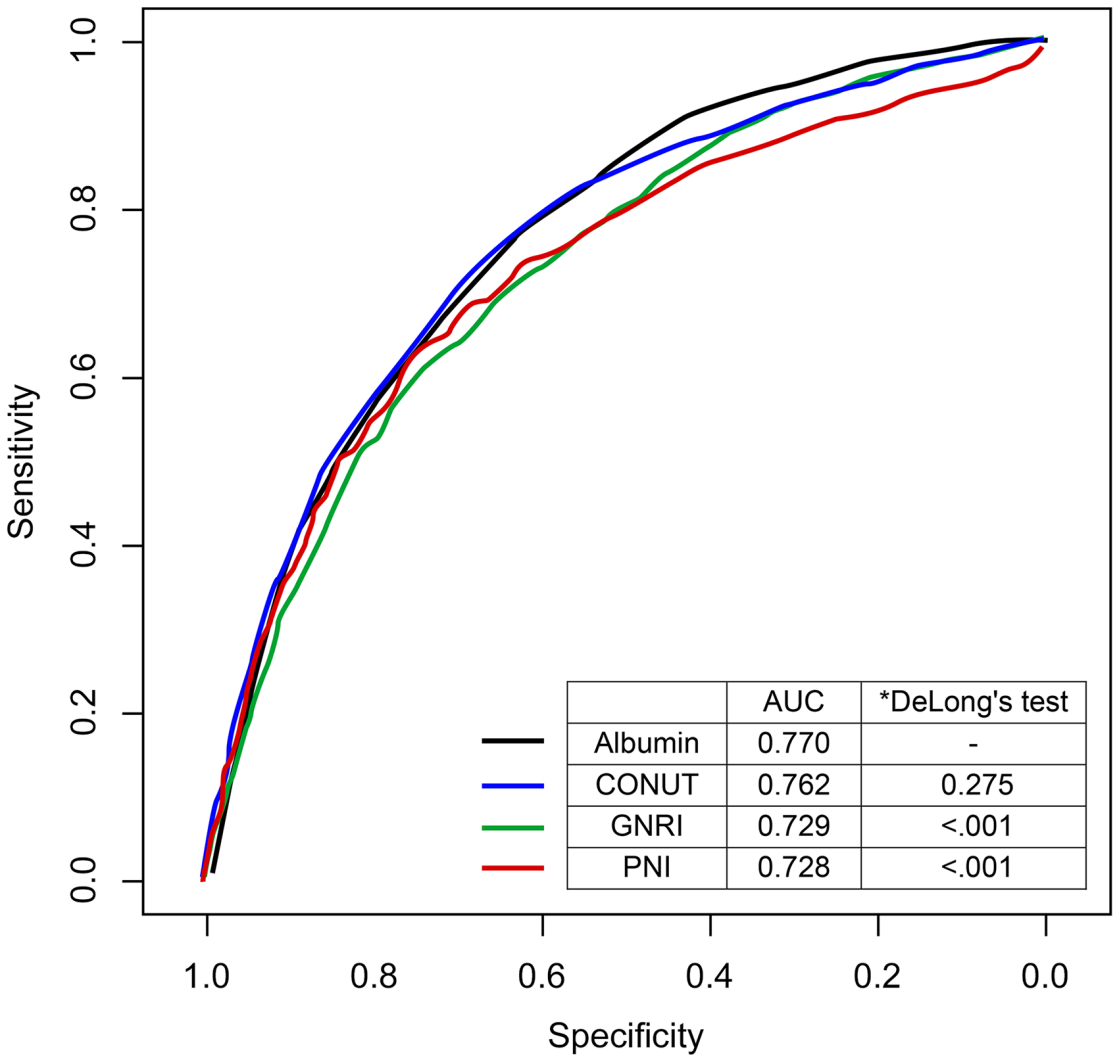
The receiver-operating characteristic curve comparing the predictive performances for delirium. The predictive performances of nutritional indices were calculated using Delong’s test and compared to those of albumin. AUC, area under the curve; CONUT, Controlling Nutritional Status; GNRI, Geriatric Nutritional Risk Index; PNI, Prognostic Nutritional Index.

The performances of albumin, PNI, GNRI, and CONUT for delirium prediction were compared and are shown in Figure 2. Among three nutritional indices, the AUROC of the CONUT was higher than those of the PNI and GNRI. Algorithm discrimination performance of albumin alone (AUC = 0.770; 95% CI, 0.750–0.790) and CONUT (AUC = 0.762; 95% CI, 0.750–0.790; *p =* 0.275) for delirium were not significantly different; however, discrimination was superior to that of the GNRI (AUC = 0.729; 95% CI, 0.706–0.753; *p <* 0.001) and PNI (AUC = 0.728; 95% CI, 0.706–0.751; *p <* 0.001).

### Factors associated with delirium

Univariable logistic regression analysis indicated that 15 characteristics of CICU patients were associated with delirium (Table 3). In multivariable analysis, age, sex, smoking, stroke, dementia, Sequential Organ Failure Assessment score, mechanical ventilation, and white blood cell were associated with increased risk of delirium, while albumin level at CICU admission (adjusted OR 0.555; 95% CI 0.402–0.767; *p* < 0.001) was associated with decreased risk of delirium.

**Table 3:** Factors associated with delirium.

	OR (95% CI)	*p*-value	^*^Adjusted OR (95% CI)	*P*-value
Age, years	1.031 (1.024–1.037)	<0.001	1.030 (1.020–1.039)	<0.001
Sex, male	1.573 (1.318–1.878)	<0.001	1.894 (1.457–2.462)	<0.001
Smoking	0.737 (0.590–0.920)	<0.001	1.602 (1.143–2.244)	0.006
Hypertension	1.386 (1.162–1.655)	<0.001		
Diabetes mellitus	1.479 (1.235–1.771)	<0.001		
Stroke	2.611 (2.077–3.282)	<0.001	2.106 (1.537–2.886)	<0.001
Dementia	8.813 (4.392–17.684)	<0.001	8.636 (3.406–21.898)	<0.001
Previous coronary disease	1.446 (1.196–1.749)	<0.001		
Chronic kidney disease^†^	1.319 (0.875–1.988)	0.186		
History of cardiac arrest	3.928 (3.044–5.069)	<0.001	1.537 (1.052–2.247)	0.026
SOFA score at admission	1.408 (1.363–1.455)	<0.001	1.335 (1.279–1.393)	<0.001
Polypharmacy^††^	0.807 (0.631–1.032)	0.088		
Mechanical ventilation	3.173 (2.649–3.799)	<0.001	1.529 (1.071–2.181)	0.019
Renal replacement therapy	0.836 (0.615–1.136)	0.253		
Intraaortic balloon pump	2.167 (1.371–3.425)	0.001		
ECMO	2.747 (2.157–3.498)	<0.001		
White blood cell count, 10^3^/μL	1.115 (1.097–1.133)	<0.001	1.058 (1.030–1.088)	<0.001
Hemoglobin, g/dL	0.727 (0.699–0.757)	<0.001		
Blood urea nitrogen, mg/dL				
Serum glucose maximum, mg/dL	1.004 (1.002–1.005)	<0.001		
Component of nutritional indices				
Albumin, g/dL	0.199 (0.169–0.234)	<0.001	0.409 (0.328–0.510)	<0.001
ALC, x10^3^/μL	0.800 (0.739–0.867)	<0.001		
Total cholesterol, mg/dL	0.986 (0.984–0.988)	<0.001		
Bodyweight/IBW	0.332 (0.189–0.583)	<0.001		

ALC, absolute lymphocyte count; CI, confidence interval; CICU, cardiac intensive care unit; IBW, ideal bodyweight; OR, odds ratio; SOFA, Sequential Organ Failure Assessment. ^*^Adjusted covariates were age, sex, smoking, hypertension, diabetes mellitus, stroke, dementia, previous coronary disease, chronic kidney disease, history of cardiac arrest, SOFA score, albumin, ALC, total cholesterol, and bodyweight/IBW. ^†^Chronic kidney disease is defined as end-stage renal disease on hemodialysis or peritoneal dialysis. ^††^Polypharmacy is defined as the routine use of five or more medications.

## Discussion

We investigated the associations of nutritional indices and their components with delirium in CICU patients from a single-center registry. The major findings were as follows: (i) nutritional indices (PNI, GNRI, and CONUT) had fair predictive performances for delirium in CICU patients; (ii) predictive performance of albumin alone for delirium was superior to those of PNI and GNRI and similar to that of CONUT; and (iii) among the components of nutritional indices, albumin was an independent predictor of delirium, whereas ALC, bodyweight/IBW, and total cholesterol were not.

Delirium is an emerging complication of CICU stay, with a reported incidence range from 5.7 to 40% (18–20). There are several possible causes for the increased incidence of delirium in CICU. First of all, acute respiratory failure caused by cardiac origin has been a leading indication for CICU admission, and recent studies have described temporal increases in mechanical ventilation support in CICU populations (21). Although the clinical practice guidelines for adult patients in the ICU recommend light sedation, some mechanically ventilated patients require deep sedation to control pain and agitation during the early phase of respiratory failure (12). For deep sedation, intravenous administration of opioids and benzodiazepines is often used, and their use is associated with delirium (22). Furthermore, the CICU has become considerably more complex with advanced medical devices, increasing CICU patient complications (3). Therefore, delirium has recently been recognized as a common comorbidity for CICU patients.

Several prospective cohort studies have evaluated the incidence of postoperative delirium in malnourished patients undergoing coronary artery bypass grafting and have found that malnutrition is associated with postoperative delirium (7, 23). Meta-analysis to determine the risk factors of delirium after orthopedic surgery in elderly patients has also shown malnutrition as an important factor for delirium after surgery (24). In CICU patients, Sugita et al. conducted a prospective cohort study to evaluate the association between malnutrition and delirium in critically ill cardiac patients (25). Of 653 CICU patients, 58 (8.9%) developed delirium, for which PNI and CONUT were both reported to be independent predictors of delirium, but GNRI was not. They suggested that ALC, included in PNI and CONUT but not in GNRI, might be an important prognostic factor associated with inflammation in delirium patients. However, in the present study, U-shaped correlation was observed between increased level of ALC and delirium. Furthermore, the discrimination performance of PNI for delirium was similar to those of CONUT and GNRI.

Albumin is the most abundant protein in the plasma and is a major transporter of many compounds in blood (26). A recent study of surgical ICU patients by Yeh DD, et al. found that serum albumin levels and pre-albumin levels may reflect baseline nutrition status. However, changes in serum albumin and pre-albumin levels during ICU stays may not indicate nutritional adequacy in the ICU (27). This is because albumin synthesis can be affected by numerous acute and chronic disease processes, and serum albumin level can provide predictive insights into the effects of disease on protein metabolism. Hypoalbuminemia can be observed in acute illness due to increased capillary leakage or decreased synthesis due to inflammation, underlying malnutrition, or hepatic synthetic dysfunction (28). Therefore, albumin is a well-known predictor of mortality in critically ill patients (29). Recent studies have reported the association between albumin level and delirium, and meta-analysis of risk factors of delirium occurrence among elderly patients in medical ICUs has found that low albumin level is associated with delirium (30). Zhang et al. analyzed 700 elderly patients admitted to the ICU after non-cardiac surgery and found that preoperative hypoalbuminemia was associated with an increased risk of postoperative delirium (31). Similarly, we found the decrease in albumin level is a risk factor of developing delirium in CICU patients. Furthermore, albumin alone revealed an inverse linear correlation with delirium, and its predictive performance was superior to those of PNI and GNRI and similar to that of CONUT. Compared with other variables included in nutritional indices, only albumin showed a linear correlation with delirium. Therefore, albumin might be a helpful and simple indicator for predicting delirium in CICU patients. This finding should be validated in future studies, and the mechanism of the relationship between albumin and delirium should be investigated further.

Although this study provides additional information on the associations of albumin and nutritional indices with delirium in CICU patients, several limitations should be noted. First, our study used nonrandomized registry data. Therefore, selection bias and confounding factors might have affected our results. Second, we evaluated delirium only using CAM-ICU. However, the CAM-ICU is generally used for delirium assessment in general ICUs, and we used this tool for routine practice in ICU management including CICU. Finally, we did not include individual information on nutritional support during CICU management collect pre albumin level for comparing albumin for nutritional status. Further studies including detailed knowledge of nutritional support would be informative.

## Conclusion

Predictive performances of nutritional indices for the occurrence of delirium were acceptable, and albumin alone as a component of nutritional indices might be a simple and helpful indicator of delirium in patients admitted to a CICU.

## Data availability statement

The raw data supporting the conclusions of this article will be made available by the authors, without undue reservation.

## Ethics statement

The studies involving human participants were reviewed and approved by the Institutional Review Board of Samsung Medical Center approved this study (IRB. No. 2020-10-102) and waived the requirement for informed consent because of the observational nature of the study.

## Author contributions

TK, RK, SN, CC, KC, C-MP, and JY contributed to conception and design of the study. TK, SN, CC, and KC organized the database. TK, RK, and JY performed the data analysis and interpretation. TK wrote the first draft of the manuscript. TK, RK, C-MP, and JY revised the manuscript. All authors contributed to manuscript revision, read, and approved the submitted version.

## Conflict of interest

The authors declare that the research was conducted in the absence of any commercial or financial relationships that could be construed as a potential conflict of interest.

## Publisher’s note

All claims expressed in this article are solely those of the authors and do not necessarily represent those of their affiliated organizations, or those of the publisher, the editors and the reviewers. Any product that may be evaluated in this article, or claim that may be made by its manufacturer, is not guaranteed or endorsed by the publisher.

## Abbreviations

ALC: absolute lymphocyte count
AUROC: area under the receiver operating characteristic curve
CAM-ICU: confusion assessment method for the intensive care unit
CI: confidence interval
CICU: cardiac intensive care unit
CONUT: Controlling Nutritional Status
GNRI: Geriatric Nutritional Risk Index
IBW: ideal bodyweight
ICU: intensive care unit
OR: odds ratio
PNI: Prognostic Nutrition Index

## References

[1] Ely EW Shintani A Truman B Speroff T Gordon SM Harrell FE Jr. Delirium as a predictor of mortality in mechanically ventilated patients in the intensive care unit. JAMA. (2004) 291:1753–62. doi: 10.1001/jama.291.14.1753 15082703

[2] Girard TD Thompson JL Pandharipande PP Brummel NE Jackson JC Patel MB. Clinical phenotypes of delirium during critical illness and severity of subsequent long-term cognitive impairment: a prospective cohort study. Lancet Respir Med. (2018) 6:213–22. doi: 10.1016/S2213-2600(18)30062-6, PMID: 29508705 PMC6709878

[3] Holland EM Moss TJ. Acute noncardiovascular illness in the cardiac intensive care unit. J Am Coll Cardiol. (2017) 69:1999–2007. doi: 10.1016/j.jacc.2017.02.033, PMID: 28427574

[4] Grotti S Falsini G. Delirium in cardiac patients. Eur Heart J. (2017) 38:2244. doi: 10.1093/eurheartj/ehx380 28810719

[5] Falsini G Grotti S Porto I Toccafondi G Fraticelli A Angioli P. Long-term prognostic value of delirium in elderly patients with acute cardiac diseases admitted to two cardiac intensive care units: a prospective study (DELIRIUM CORDIS). Eur Heart J Acute Cardiovasc Care. (2018) 7:661–70. doi: 10.1177/2048872617695235, PMID: 29064263

[6] Ritchie C Walters RW Ramaswamy S Alla VM. Impact of delirium on mortality in patients hospitalized for heart failure. Int J Psychiatry Med. (2021) 57:212–25. doi: 10.1177/00912174211028019 34176306

[7] Ringaitienė D Gineitytė D Vicka V Žvirblis T Šipylaitė J Irnius A. Impact of malnutrition on postoperative delirium development after on pump coronary artery bypass grafting. J Cardiothorac Surg. (2015) 10:74. doi: 10.1186/s13019-015-0278-x, PMID: 25990791 PMC4449612

[8] Buzby GP Mullen JL Matthews DC Hobbs CL Rosato EF. Prognostic nutritional index in gastrointestinal surgery. Am J Surg. (1980) 139:160–7. doi: 10.1016/0002-9610(80)90246-9 7350839

[9] Bouillanne O Morineau G Dupont C Coulombel I Vincent JP Nicolis I. Geriatric nutritional risk index: a new index for evaluating at-risk elderly medical patients. Am J Clin Nutr. (2005) 82:777–83. doi: 10.1093/ajcn/82.4.777, PMID: 16210706

[10] Ignacio de Ulibarri J Gonzalez-Madrono A de Villar NG Gonzalez P Gonzalez B Mancha A. CONUT: a tool for controlling nutritional status. First validation in a hospital population. Nutr Hosp. (2005) 20:38–45. PMID: 15762418

[11] Na SJ Chung CR Jeon K Park CM Suh GY Ahn JH. Association between presence of a cardiac intensivist and mortality in an adult cardiac care unit. J Am Coll Cardiol. (2016) 68:2637–48. doi: 10.1016/j.jacc.2016.09.947, PMID: 27978948

[12] Devlin JW Skrobik Y Gelinas C Needham DM Slooter AJC Pandharipande PP. Clinical practice guidelines for the prevention and Management of Pain, agitation/sedation, delirium, immobility, and sleep disruption in adult patients in the ICU. Crit Care Med. (2018) 46:e825–73. doi: 10.1097/CCM.0000000000003299, PMID: 30113379

[13] Alvares-da-Silva MR Reverbel da Silveira T. Comparison between handgrip strength, subjective global assessment, and prognostic nutritional index in assessing malnutrition and predicting clinical outcome in cirrhotic outpatients. Nutrition. (2005) 21:113–7. doi: 10.1016/j.nut.2004.02.002, PMID: 15723736

[14] Li W Li M Wang T Ma G Deng Y Pu D. Controlling nutritional status (CONUT) score is a prognostic factor in patients with resected breast cancer. Sci Rep. (2020) 10:6633. doi: 10.1038/s41598-020-63610-7, PMID: 32313183 PMC7171067

[15] Colantuoni E Dinglas VD Ely EW Hopkins RO Needham DM. Statistical methods for evaluating delirium in the ICU. Lancet Respir Med. (2016) 4:534–6. doi: 10.1016/S2213-2600(16)30138-2 27264776 PMC13730309

[16] Pazan F Wehling M. Polypharmacy in older adults: a narrative review of definitions, epidemiology and consequences. Eur Geriatr Med. (2021) 12:443–52. doi: 10.1007/s41999-021-00479-3, PMID: 33694123 PMC8149355

[17] DeLong ER DeLong DM Clarke-Pearson DL. Comparing the areas under two or more correlated receiver operating characteristic curves: a nonparametric approach. Biometrics. (1988) 44:837–45. doi: 10.2307/2531595 3203132

[18] Pauley E Lishmanov A Schumann S Gala GJ van Diepen S Katz JN. Delirium is a robust predictor of morbidity and mortality among critically ill patients treated in the cardiac intensive care unit. Am Heart J. (2015) 170:79–86.e1. doi: 10.1016/j.ahj.2015.04.013, PMID: 26093867

[19] Ibrahim K McCarthy CP McCarthy KJ Brown CH Needham DM Januzzi JL Jr. Delirium in the cardiac intensive care unit. J Am Heart Assoc. (2018) 7:e008568. doi: 10.1161/JAHA.118.008568, PMID: 29453307 PMC5850211

[20] Chang YL Tsai YF Lin PJ Chen MC Liu CY. Prevalence and risk factors for postoperative delirium in a cardiovascular intensive care unit. Am J Crit Care. (2008) 17:567–75. doi: 10.4037/ajcc2008.17.6.567, PMID: 18978241

[21] Jentzer JC van Diepen S Barsness GW Katz JN Wiley BM Bennett CE. Changes in comorbidities, diagnoses, therapies and outcomes in a contemporary cardiac intensive care unit population. Am Heart J. (2019) 215:12–9. doi: 10.1016/j.ahj.2019.05.012, PMID: 31260901

[22] Pisani MA Murphy TE Araujo KL Slattum P Van Ness PH Inouye SK. Benzodiazepine and opioid use and the duration of intensive care unit delirium in an older population. Crit Care Med. (2009) 37:177–83. doi: 10.1097/CCM.0b013e318192fcf9, PMID: 19050611 PMC2700732

[23] Velayati A Vahdat Shariatpanahi M Shahbazi E Vahdat SZ. Association between preoperative nutritional status and postoperative delirium in individuals with coronary artery bypass graft surgery: a prospective cohort study. Nutrition. (2019) 66:227–32. doi: 10.1016/j.nut.2019.06.006, PMID: 31357095

[24] Yang Y Zhao X Gao L Wang Y Wang J. Incidence and associated factors of delirium after orthopedic surgery in elderly patients: a systematic review and meta-analysis. Aging Clin Exp Res. (2021) 33:1493–506. doi: 10.1007/s40520-020-01674-1, PMID: 32772312

[25] Sugita Y Miyazaki T Shimada K Shimizu M Kunimoto M Ouchi S. Correlation of nutritional indices on admission to the coronary intensive care unit with the development of delirium. Nutrients. (2018) 10:1712. doi: 10.3390/nu10111712, PMID: 30413062 PMC6267104

[26] Fanali G di Masi A Trezza V Marino M Fasano M Ascenzi P. Human serum albumin: from bench to bedside. Mol Asp Med. (2012) 33:209–90. doi: 10.1016/j.mam.2011.12.002, PMID: 22230555

[27] Yeh DD Johnson E Harrison T Kaafarani HMA Lee J Fagenholz P. Serum levels of albumin and Prealbumin do not correlate with nutrient delivery in surgical intensive care unit patients. Nutr Clin Pract. (2018) 33:419–25. doi: 10.1002/ncp.10087, PMID: 29665145

[28] Soeters PB Wolfe RR Shenkin A. Hypoalbuminemia: pathogenesis and clinical significance. JPEN J Parenter Enteral Nutr. (2019) 43:181–93. doi: 10.1002/jpen.1451, PMID: 30288759 PMC7379941

[29] Knaus WA Wagner DP Draper EA Zimmerman JE Bergner M Bastos PG. The APACHE III prognostic system. Risk prediction of hospital mortality for critically ill hospitalized adults. Chest. (1991) 100:1619–36. doi: 10.1378/chest.100.6.1619 1959406

[30] Ahmed S Leurent B Sampson EL. Risk factors for incident delirium among older people in acute hospital medical units: a systematic review and meta-analysis. Age Ageing. (2014) 43:326–33. doi: 10.1093/ageing/afu022, PMID: 24610863 PMC4001175

[31] Zhang DF Su X Meng ZT Cui F Li HL Wang DX. Preoperative severe hypoalbuminemia is associated with an increased risk of postoperative delirium in elderly patients: results of a secondary analysis. J Crit Care. (2018) 44:45–50. doi: 10.1016/j.jcrc.2017.09.182, PMID: 29055835

